# Rethinking the Action Observation and Execution Network After Early Brain Damage: A Developmental Framework for Cerebral Palsy

**DOI:** 10.3390/children13101303

**Published:** 2026-09-25

**Authors:** Eleonora Sicuri, Giuseppe Ciullo, Donatella Saviola, Antonio De Tanti, Antonino Errante

**Affiliations:** 1 Department of Medicine and Surgery, University of Parma, 43125 Parma, Italy; eleonora.sicuri@unipr.it (E.S.); giuseppe.ciullo@unipr.it (G.C.); 2 Neuroradiology Unit, Fondazione IRCCS Istituto Neurologico Carlo Besta, 20133 Milano, Italy; 3 Centro Cardinal Ferrari KOS, 43012 Parma, Italy; donatella.saviola@centrocardinalferrari.it (D.S.); antonio.detanti@kosgroup.com (A.D.T.)

**Keywords:** action observation treatment, cerebral palsy, action observation and execution network, rehabilitation, brain damage

## Abstract

**Highlights:**

**What are the main findings?**

- Children with cerebral palsy show functionally responsive action observation–execution mechanisms, but early brain lesions, network reorganization, and atypical motor experience may alter the neural representation of action goals and kinematics.
- Motor repertoire and neuropsychological functions—including attention, praxis, visuospatial processing, working memory, and executive control—may contribute to individual variability in action observation and execution network recruitment and response to action observation treatment.

**What are the implications of the main findings?**

- Future action observation treatment approaches may benefit from moving beyond standardized protocols and considering the child’s structural and functional brain organization, motor phenotype, and neuropsychological profile.
- Multimodal phenotyping may inform the selection of the observed model, task complexity, presentation format, and level of cognitive support according to individual characteristics.

**Abstract:**

**Background/Objectives**: The action observation and execution network (AOEN) is thought to support action perception–execution coupling and it may contribute to motor learning during development. In cerebral palsy (CP), early brain lesions occur while sensorimotor and cognitive networks are still developing, potentially reshaping AOEN organization and the mechanisms through which children learn from observed actions. This narrative review examines current evidence on AOEN involvement during action observation and execution in CP and proposes a neuroconstructivist framework for personalized observation-based rehabilitation. **Methods**: We synthesized behavioural, neuroimaging, and intervention studies investigating AOEN functioning, structural and functional reorganization, and Action Observation Treatment (AOT) in children with CP, with particular attention to unilateral and bilateral phenotypes. **Results**: Available evidence suggests that action observation–execution mechanisms remain functionally responsive in CP, although their organization may be altered according to lesion architecture, distributed network connectivity, and acquired motor experience. Motor impairment may influence the encoding of kinematics, goals, and action sequences, while models whose movements are closer to the child’s motor repertoire have been associated with greater neural engagement and preliminary short-term treatment benefits. Praxis, attention, working memory, visuospatial processing, and executive functions may further influence the transformation of observed actions into executable motor programmes. **Conclusions**: We propose that rehabilitation response may reflect the interaction among structural and functional reorganization, motor phenotype, neuropsychological functioning, environmental experience, and stimulus characteristics. The AOEN may therefore be better conceptualized as a developmentally reconfigured network rather than a uniformly preserved or impaired system. Multimodal phenotyping may help support more developmentally informed and individualized observation-based rehabilitation.

## 1. Overview

The discovery of the mirror mechanism substantially changed the traditional view of the motor system as a neural architecture devoted exclusively to movement execution [1]. Building on this framework, a broader action observation and execution network (AOEN), centred on premotor and inferior parietal regions and interacting with somatosensory, visual, basal ganglia, thalamic, and cerebellar circuits, is recruited both when an individual performs an action and when that action is perceived by others [1]. Within this network, some regions appear to be preferentially engaged by observation, such as the posterior superior temporal sulcus and extrastriate visual areas, whereas others are more selectively recruited during execution, such as primary motor and somatosensory cortices. A core set of regions, including ventral premotor cortex, inferior frontal gyrus, and inferior parietal lobule, is consistently activated during both observation and execution and constitutes the shared frontoparietal component historically associated with the mirror mechanism. This perception–action coupling is thought to support the representation of movement kinematics, immediate motor goals, and action intentions, thereby contributing to imitation, motor learning, and social interaction [1,2,3,4,5]. Developmental evidence further indicates that neural responses within the AOEN are already detectable early in life but continue to specialize through childhood as a function of maturation and sensorimotor experience [6]. The AOEN can therefore be conceptualized not as a fixed module, but as an experience-dependent network whose organization reflects the motor repertoire that the child has been able to acquire. In the present context, the term motor repertoire refers to the motor modules available in the child’s spontaneous motor production. These modules represent individual motor elements that, when combined, give rise to meaningful postures and gestures. Motor repertoire can therefore be characterized both quantitatively, in terms of the amount of motor production, and qualitatively, in terms of the variability and types of movements expressed [7].

This developmental perspective is particularly relevant to cerebral palsy (CP), a heterogeneous group of permanent disorders of movement and posture caused by non-progressive disturbances of the developing fetal or infant brain and frequently accompanied by sensory, cognitive, communicative, behavioural, and epileptic comorbidities [8]. Given that the AOEN develops in close interaction with these same sensorimotor and cognitive systems, it is plausible that its functioning is also modulated, rather than uniformly disrupted, in children with CP. Consistent with this view, children with CP have been shown to recruit the AOEN atypically, displaying patterns of network organization that differ from those observed in typically developing peers [9]. Such findings suggest that the AOEN in CP remains functionally responsive, but its recruitment may be shaped by the child’s own sensorimotor and cognitive profile.

In CP, the early lesion does not simply remove function from an otherwise mature system; rather, it alters the developmental trajectory through which corticospinal, thalamocortical, frontoparietal, and cortico-subcortical-cerebellar circuits are selected and refined. Lesion timing, location, and extent, as well as the resulting pattern of ipsilesional or contralesional motor reorganization, can therefore shape both the execution and perception of actions. A recent lesion-mapping study from our lab has shown that distinct manipulative profiles in unilateral CP are associated with different lesion distributions and patterns of cortical and subcortical involvement, supporting the view that apparently similar motor phenotypes may arise from different neurobiological substrates [10]. Because the anatomical substrate available to support action observation differs across children, variability in AOEN function, and in the capacity to benefit from observation-based learning, may therefore represent an intrinsic feature of the condition rather than methodological noise.

Nevertheless, available neurophysiological and neuroimaging studies indicate that the AOEN is present in children with CP. Its organization is not simply reduced, but reconfigured: its activity may be more bilateral or preferentially supported by the spared hemisphere, and its response may depend on the child’s previous motor experience and on the kinematic characteristics of the observed action [9,11,12,13]. In an fMRI study from our group, children with unilateral CP (UCP) showed stronger AOEN engagement when observing actions performed by a pathological ameliorative model whose movement kinematics were closer to their own motor repertoire than when observing a typically developing model [12]. These preliminary neuroimaging findings subsequently motivated a randomized controlled trial showing positive effects of AOT using a pathological ameliorative model, although replication of the findings is still needed [11]. Complementary evidence that these children can generate explicit motor representations of grasping actions further supports the presence, albeit atypical organization, of internal action simulation mechanisms [14]. Together, these findings provide a neurobiological rationale for rehabilitation based on systematic action observation: the relevant network appears to remain functionally responsive and potentially recruitable even when active movement is constrained.

Action Observation Treatment (AOT), in which goal-directed actions are systematically observed and subsequently imitated, was developed to exploit this neurophysiological mechanism and strengthen motor representations through repeated perception–execution coupling [15] (Figure 1). Clinical trials in children with CP have reported improvements in upper-limb activity and manual function, accompanied in some studies by changes in parieto-premotor activation or sensorimotor mu-rhythm modulation [16,17,18]. However, this evidence remains heterogeneous, and recent meta-analyses have emphasized imprecision, small samples, and uncertainty regarding the superiority of AOT over control observation or other active therapies [19,20,21]. Rather than undermining the mechanistic rationale for AOT, this heterogeneity suggests that treatment response may vary according to individual clinical and neurobiological characteristics, as well as to the features of the observed model. Consistent with this interpretation, a randomized trial from our lab found that an AOT protocol based on pathological but improvable kinematics was associated with greater short-term upper-limb gains than observation of a typically developing model, together with treatment-related modulation of ventral premotor activity [18].

A further source of heterogeneity is the child’s neuropsychological profile. Executive dysfunction, attentional control difficulties, visuospatial deficits, praxis disorders, learning problems, and behavioural or emotional symptoms are common in CP and are related to the distribution of brain damage rather than being secondary features of the motor disorder [22,23,24,25,26]. These functions are directly implicated in the processes required by AOT: maintaining attention to the model, selecting relevant kinematic cues, retaining an action sequence in working memory, inhibiting ineffective responses, forming an action plan, and translating the observed movement into goal-directed execution. Neuropsychological functioning may therefore moderate both AOEN recruitment and treatment adherence, learning rate, generalization, and functional outcome. From this perspective, neuropsychological assessment may represent an important component of motor rehabilitation, helping guide decisions regarding task complexity, cueing, session duration, model selection, and the degree of therapist or caregiver scaffolding [27].

Taken together, the present review integrates four levels of evidence that are often considered separately in the current literature: the developmental neurobiology of the AOEN; structural and functional reorganization after early brain lesions; behavioural, electrophysiological, and neuroimaging evidence for action observation–execution coupling in CP; and the clinical literature on AOT. We then examine how lesion architecture and neuropsychological functioning may interact with motor impairment to shape AOEN organization and rehabilitation response. Building on this synthesis, we propose a child-centred framework in which observation-based treatment may be individualized according to the individual combination of lesion pattern, motor repertoire, and cognitive–behavioural profile. Such an approach moves beyond asking whether AOT works and instead addresses the more clinically informative question of which observation-based treatment characteristics may be most appropriate for individual children.

The literature discussed in this review was identified through a narrative search of PubMed, Scopus, and Web of Science, focusing on studies addressing action observation, action execution, cerebral palsy, neurodevelopment, neuroimaging, electrophysiology, motor learning, and Action Observation Treatment. Particular emphasis was placed on studies involving children with unilateral or bilateral CP, together with developmental and mechanistic studies relevant to AOEN maturation and reorganization. Additional references were identified from the reference lists of key primary studies and reviews. The search covered database inception to July 2026; study selection was guided by relevance to the developmental, mechanistic, and rehabilitative aims of this narrative review.

## 2. Developmental Neurobiology of the AOEN

The AOEN comprises distributed frontoparietal regions involved in mapping observed actions onto the observer’s own motor representations [1]. Evidence accumulated over the last two decades suggests that the AOEN is not a static system fully mature at birth, but rather a dynamic network that progressively emerges and specializes through the interaction between biological predispositions, brain maturation, and sensorimotor experience [28,29]. Developmental models propose that the functional properties of the AOEN are shaped through experience-dependent plasticity, as the repeated temporal co-occurrence of action execution and observation strengthens perception–action links and progressively refines shared action representations [30,31].

Within this framework, early sensorimotor experience plays a crucial role in establishing the neural mechanisms through which observed actions are internally represented and understood. Consistent with this view, Gallese and colleagues [32] proposed that embodied sensorimotor processes provide a basis for action understanding, while developmental perspectives emphasize that the infant’s own motor repertoire constrains and guides the perception of others’ actions. Converging evidence from electroencephalography (EEG) and functional near-infrared spectroscopy (fNIRS) indicates that neural responses to action observation are already detectable during the first months of life and involve central, frontal, and parietal regions broadly consistent with the AOEN described in adults [6]. These early responses, however, should not be interpreted as evidence of a fully mature AOEN. Rather, perception–action coupling appears to undergo substantial refinement throughout childhood and adolescence [33].

Neural markers associated with action observation, including sensorimotor mu-rhythm modulation, become increasingly differentiated and selective with age, reflecting the progressive specialization of the networks involved in action representation. Although mu-rhythm suppression is not a specific or direct measure of AOEN activity, its modulation during both action observation and execution provides useful evidence of shared sensorimotor processing. The use of mu-rhythm as a marker in children with CP, however, presents specific limitations. Structural brain lesions and abnormal cortical organization can alter EEG signal propagation and topography. Accordingly, children with CP have been reported to show atypical mu-rhythm topography, reduced desynchronization over the lesioned hemisphere, and considerable variability across individuals [34]. Moreover, lesion-related anatomical asymmetries and volume-conduction effects may influence scalp topography and apparent hemispheric lateralization, which should therefore not be interpreted as a direct map of cortical AOEN organization. Data in this clinical population remain scarce, and it is not yet known whether mu-rhythm patterns are consistent within the same child over time. These limitations suggest that mu-rhythm findings in CP should be interpreted with caution as evidence regarding AOEN function. This developmental trajectory parallels improvements in motor control, action prediction, imitation, and social cognition, suggesting that AOEN maturation is closely intertwined with broader cognitive and motor development [35,36]. Importantly, infant studies also indicate that action observation responses are influenced by prior motor experience and by whether the observed action is already included in the child’s motor repertoire, supporting an early experience-dependent organization of perception–action coupling [6].

A central aspect of this process concerns the emergence of imitation abilities. Early imitation has been proposed as one behavioural manifestation of perception–action coupling, allowing infants to translate observed actions into corresponding motor outputs [37,38]. Whether neonates are actually capable of imitation, however, is still debated. Some researchers argue that early reports of neonatal imitation reflect methodological artefacts rather than a genuine capacity present at birth [39], while others maintain that neonatal imitation is a real phenomenon, at least for some gestures such as tongue protrusion [40]. This controversy has broader implications for theories that assume an innate, foundational role of imitation in early social cognitive development. Whether imitation is present from birth or emerges through early postnatal experience, it may represent an important mechanism through which sensorimotor associations are reinforced and the AOEN progressively organized. In turn, increasingly refined motor representations facilitate more accurate action perception, supporting a bidirectional relationship between acting and observing [32].

Additionally, motor development appears to co-develop with language acquisition, suggesting that sensorimotor maturation may provide a scaffold not only for action understanding but also for the emergence of communicative abilities. Several theoretical perspectives propose that language and action systems share partially overlapping neural substrates and developmental mechanisms grounded in bodily experience rather than exclusively in amodal representations [41]. Within this perspective, the mouth represents a particularly relevant interface between bodily experience and early cognition: beyond its role in speech production, it contributes to tactile exploration and social interaction, allowing infants to link sensorimotor experiences with emerging communicative and conceptual representations. The progressive development of orofacial and manual skills may therefore contribute to both action understanding and the scaffolding of linguistic abilities. This view is consistent with broader embodied-cognition accounts, according to which conceptual and linguistic representations are grounded, at least in part, in sensorimotor experience [42,43].

Development throughout childhood and adolescence is accompanied by marked structural and functional changes in cortical regions associated with the AOEN. Longitudinal neurodevelopmental studies have shown that frontal and parietal areas involved in action observation and motor planning undergo prolonged maturation extending into adolescence [44,45]. This cortical remodelling is accompanied by changes in both the extent and functional organization of the AOEN. Functional MRI evidence indicates that children already recruit a cortical network largely overlapping with that observed in adults, including the premotor cortex, posterior inferior frontal gyrus, and posterior parietal cortex [46]. However, activation during childhood is generally more widespread, more bilateral, and more variable across individuals, suggesting that action representations are initially supported by a relatively diffuse and less specialized neural architecture. With increasing age, activation becomes more focal and the network shows progressively greater left-hemispheric lateralization, particularly during the observation of complex hand actions.

Importantly, the transition from childhood to adolescence does not appear to involve only a linear reduction in activation. In a longitudinal fMRI study, Shaw and colleagues [47] identified non-linear, quadratic developmental trajectories within frontoparietal regions of the AOEN during early adolescence. These findings suggest a phase of functional reorganization in which neural recruitment may temporarily increase or change before reaching a more stable adult-like configuration [48]. Consistent with this interpretation, Lesourd and colleagues [49] found that adolescents and adults recruited broadly comparable frontoparietal and occipitotemporal regions while observing object-directed and socially directed actions. However, multivariate analyses revealed less robust representations of action sociality and transitivity in posterior parietal regions in adolescents, together with reduced generalization of social action representations in the lateral occipitotemporal cortex. Thus, although the overall architecture of the AOEN is already established during adolescence, its fine-grained coding of action content—particularly its social dimension—continues to mature.

Taken together, current developmental evidence supports the view that the AOEN develops through a prolonged process of experience-dependent plasticity integrating motor experience, imitation, communication, and social learning. Through maturation and repeated sensorimotor interactions, the system progressively develops into a hierarchical and partially lateralized network capable of representing multiple levels of action information, from movement kinematics to goals and intentions. This normative developmental trajectory provides an essential reference for understanding how early brain lesions may affect the AOEN differently depending on the developmental stage at which they occur. Rather than disrupting a fully established system, early injury may interfere with ongoing processes of network specialization, lateralization, and experience-dependent refinement, thereby redirecting subsequent perception–action development.

## 3. Early Brain Lesions and Neuroplastic Reorganization in Cerebral Palsy

The consequences of an early brain lesion depend on the developmental stage at which the insult occurs, but the traditional assumption that “the younger the age at insult, the better the outcome” has progressively been replaced by a more complex view of developmental neuroplasticity [50,51]. Lesion timing, location, extent, unilateral or bilateral distribution, and subsequent sensorimotor experience interact in shaping the outcome. In the case of the AOEN, however, the relationship between lesion timing and specific stages of network development remains incompletely understood, because its normative developmental trajectory has itself not yet been fully characterized. Available evidence nevertheless indicates that the AOEN undergoes prolonged maturation and experience-dependent refinement. Early brain injury may therefore interact with different phases of this developmental process, potentially redirecting rather than simply disrupting the emerging network. Importantly, lesion timing should not be interpreted as a deterministic predictor of the resulting AOEN organization. Injuries occurring within the same developmental period may differ substantially in their anatomical targets and network consequences, while lesions arising at different stages may converge on partially similar patterns of functional reorganization. Congenital malformations arising early in development may influence the formation and connectivity of cortical and subcortical components of the AOEN, whereas later prenatal or perinatal injuries may directly damage emerging cortical nodes or their connections. Thus, the consequences for AOEN development are likely to depend on the interaction between when the injury occurs and which nodes and pathways are affected, rather than on timing alone. Early plasticity is therefore not intrinsically adaptive: the same developmental mechanisms that permit compensation may also support maladaptive patterns of neural organization.

Neuroimaging studies have identified periventricular white-matter injury, cortical and deep-grey-matter lesions, focal infarctions, and cerebral malformations as the main structural patterns associated with CP [52,53]. Periventricular lesions generally occur earlier and may affect corticospinal and thalamocortical fibres, whereas lesions occurring closer to term more often involve cortical, subcortical, and deep-grey-matter structures. These categories remain clinically useful, but they do not fully explain the marked variability of motor phenotypes within each lesion type. From a network perspective, functional outcome may depend not only on the anatomical location of the lesion itself, but also on the extent to which it disrupts developing cortical and subcortical connections.

A major determinant of this variability is the developmental reorganization of the corticospinal system. During typical development, corticospinal projections are initially bilateral, ipsilateral projections are progressively withdrawn, and contralateral projections are strengthened through activity-dependent competition [54,55]. Following congenital brain damage, motor control of the affected hand may remain within the ipsilesional hemisphere through spared primary or non-primary motor regions, or may shift toward the contralesional hemisphere through persistent ipsilateral projections [56,57,58,59,60]. Staudt and colleagues showed that smaller periventricular lesions more often preserve contralateral corticospinal projections from the affected hemisphere, whereas larger lesions are more frequently associated with ipsilateral control from the unaffected hemisphere. Although corticospinal organization does not directly index AOEN function, these alternative developmental motor pathways may substantially alter the sensorimotor experience through which observation–execution associations are established and refined.

These classical models remain fundamental, but contemporary evidence indicates that corticospinal wiring alone is insufficient to explain motor function. The work of Mailleux, Feys, Ortibus and colleagues [61,62,63,64] has shown that upper-limb impairment in UCP reflects the combined contribution of lesion extent, corticospinal organization, somatosensory pathways, thalamocortical radiations, and transcallosal connectivity. Preserved contralateral corticospinal projections are generally associated with better motor function, whereas ipsilateral or bilateral wiring, greater lesion burden, and involvement of the posterior limb of the internal capsule, basal ganglia, and thalamus are associated with more severe motor and sensory deficits. Diffusion MRI studies further demonstrate that unimanual capacity and bimanual performance depend on the integrity and interhemispheric balance of distributed motor, sensory, and callosal pathways rather than on a single tract [65,66]. Diffusion-derived measures such as fractional anisotropy can provide indirect indices of the microstructural organization of these pathways. Reduced integrity of distributed sensorimotor and interhemispheric connections could therefore plausibly constrain the interactions through which AOEN function develops; however, direct tract-specific correlations between diffusion abnormalities and AOEN functional responses in children with CP remain scarce. These findings support a network-level view of developmental reorganization, in which structural disconnection may influence not only motor output but also the distributed sensorimotor interactions through which AOEN function develops.

Evidence obtained using clinically applicable structural MRI measures further supports a measurable relationship between lesion characteristics and manual function. Beani and colleagues [67] found moderate-to-strong associations between semiquantitative MRI lesion scores and manual ability and gross dexterity in children with UCP, with greater hemispheric, basal-ganglia, callosal, and overall lesion burden associated with poorer performance, particularly in the more affected hand. This network-based account is particularly relevant for understanding qualitative differences in hand use. Another study from our lab [10] showed that children with hemiplegic CP displaying different patterns of object manipulation were characterized by distinct combinations of cortical, subcortical, and white-matter damage. More constrained synergistic manipulation was associated with greater subcortical involvement and disruption of relevant white-matter connections, whereas semi-functional manipulation patterns showed different structural and functional profiles. Importantly, lesion location was more informative than broad lesion timing in distinguishing manipulation phenotypes. Thus, lesion analysis should not be limited to predicting the severity of motor impairment but should also address how grasping and object manipulation are organized, and how efficiently these motor patterns support functional performance.

The frequently reported observation that children with apparently similar lesions show different functional outcomes should therefore not be interpreted as evidence of a weak relationship between brain structure and behaviour. Rather, it reflects the limitations of broad qualitative lesion classifications and visual inspection of conventional MRI. Lesions that appear similar may have very different functional consequences depending on whether they interrupt corticospinal, thalamocortical, corticostriatal, or transcallosal connections, and on how spared pathways reorganize. Modern lesion mapping, diffusion imaging, tractography, and neurophysiology now allow these patterns of damage, disconnection, and rewiring to be studied quantitatively within a network-based framework. Within this perspective, the functional consequences of a lesion for the AOEN are better understood in terms of affected nodes, disrupted connections, and subsequent developmental reorganization than by lesion timing alone.

Evidence remains considerably more limited for bilateral phenotypes of CP, including spastic diplegia, and for dyskinetic or mixed phenotypes. This imbalance largely reflects the fact that neuroimaging studies of action observation, upper-limb organization, and corticospinal reorganization have predominantly focused on children with UCP. Available studies suggest that white-matter abnormalities in bilateral CP extend beyond primary motor pathways and may involve callosal, thalamocortical, and distributed sensorimotor networks. However, the relationships between lesion pattern, structural disconnection, upper-limb function, and action observation mechanisms in these populations remain poorly characterized. Accordingly, any proposed relationship between bilateral lesion patterns and AOEN reorganization should currently be considered a working hypothesis rather than an established neurofunctional profile.

Structural reorganization may also contribute to variability in rehabilitation outcomes. The capacity to benefit from motor learning and action observation-based interventions is likely to depend on the residual integrity of motor, sensory, interhemispheric, and cortico-subcortical pathways. At present, available evidence does not support the use of any single neuroimaging marker as a sufficiently validated predictor of individual treatment response in children with CP. Rather, lesion topography, structural connectivity, functional activations, corticospinal organization, and motor phenotype may represent potential components of an individualized rehabilitation framework. Taken together, contemporary evidence indicates that early brain injury initiates a developmental process of network reorganization whose final configuration contributes substantially to the child’s motor phenotype. Similar lesions may lead to different outcomes as they produce different patterns of disconnection and compensatory wiring. Characterizing this structural organization is therefore essential for understanding individual variability and developing biologically informed models of rehabilitation response [68].

## 4. Evidence of Mechanisms for Action Observation/Execution in Children with CP

The neural and behavioural mechanisms supporting action observation and execution in children with CP have been investigated using functional neuroimaging, electrophysiology, and clinical intervention paradigms. Early functional imaging studies showed that even the observation of simple hand movements recruits motor-related cortical regions in children with UCP. For example, Dinomais and colleagues [11] reported bilateral activation across frontoparietal regions associated with the AOEN during the observation of repetitive hand movements, together with modulation of primary motor cortex activity according to the observed hand. A complementary study showed that simultaneous observation and passive mobilization of the same movement produced partially overlapping motor activations, consistent with the recruitment of shared sensorimotor representations even when active execution is limited [69]. Similarly, Van de Winckel and colleagues [70] found that both active and robot-assisted passive finger movements recruited sensorimotor regions, with passive movement producing additional somatosensory engagement. Together, these findings indicate that sensorimotor networks in UCP remain responsive to multiple forms of visual and proprioceptive input, although such activation should not be interpreted as direct evidence of mirror-neuron activity or of preserved AOEN function as a whole.

More complex paradigms suggest that early motor impairment alters the way observed actions are represented. Sgandurra and colleagues [9] examined the observation of simple and complex object-manipulation actions performed with either hand. Compared with typically developing children, the UCP group showed greater lateralization of the AOEN toward the unaffected hemisphere and no clear modulation according to action complexity. This finding may indicate reduced differentiation of kinematic demands, altered allocation of neural resources, or a stronger weighting of action goals over detailed movement parameters. However, these interpretations remain indirect, because differences in activation do not by themselves establish whether the network preferentially encodes action goals, movement kinematics, or other task-related features. Thus, the consequences of early brain damage may concern not only the magnitude of sensorimotor and AOEN-related recruitment, but also the way in which different levels of action information are represented within the network. Functional MRI findings in children with CP also require specific methodological caution. Most available studies include relatively small and clinically heterogeneous samples, while head motion represents a particular concern in paediatric and motor-impaired populations. In addition, lesion-related anatomical abnormalities may affect spatial normalization, segmentation, and interindividual anatomical correspondence, potentially influencing group-level estimates of activation. Differences in vascular or haemodynamic responses after early brain injury may provide an additional source of variability in the BOLD signal. Therefore, differences in the magnitude or lateralization of fMRI activation should be interpreted as indirect measures of network recruitment rather than as direct indices of AOEN integrity or efficiency.

Electrophysiological findings broadly converge with this interpretation. Jongsma and colleagues [13] reported mu-rhythm modulation during action execution, observation, and motor imagery predominantly over the spared hemisphere in children with UCP, suggesting an altered balance of sensorimotor engagement. However, the degree and direction of lateralization should not be considered uniform across children, as they are likely influenced by lesion characteristics, corticospinal organization, residual motor function, and the specific task used. As discussed above, these electrophysiological findings should be interpreted cautiously and as indirect evidence of AOEN-related sensorimotor processing.

The evidence that action observation–execution coupling remains functionally recruitable in children with CP provides a neurobiological rationale for AOT. In an early randomized controlled trial, Sgandurra and colleagues [16] showed that children with UCP who observed and subsequently executed unimanual and bimanual daily-life actions achieved greater improvements in upper-limb function than children who performed comparable motor practice after viewing non-biological visual stimuli. Subsequent studies extended these findings and indicated that behavioural improvement may be accompanied by functional changes within motor-related cortical networks [17,71,72,73]. Buccino and colleagues [17], for example, reported improved upper-limb performance after AOT in children with CP, together with increased post-treatment recruitment of parieto-premotor regions during object manipulation. Intervention-based neuroimaging studies have similarly shown that reorganized sensorimotor networks in UCP remain susceptible to experience-dependent modification, suggesting that early reorganization does not eliminate plasticity but may influence the neural pathways through which subsequent motor learning is supported [74]. These converging behavioural and neuroimaging findings are consistent with the possibility that AOT-related functional reorganization contributes to rehabilitation gains, although the specific neural changes mediating individual treatment response remain to be established.

An important aspect concerns the relationship between the observed action and the child’s own motor repertoire. Experimental evidence in typical development indicates that AOEN-related sensorimotor recruitment is shaped by prior motor experience and by the observer’s ability to perform the observed action. In UCP, reduced and atypical experience with the affected hand may therefore influence how actions performed by typically developing models are represented. In a previous work [12], our lab directly examined this hypothesis by comparing the observation of grasping actions performed by a typically developing hand with actions performed by a model with motor impairment, whose movement kinematics were more similar to those experienced by children with UCP. Observation of both models recruited the AOEN bilaterally, although activation was more strongly lateralized toward the contralesional hemisphere in the UCP group. Crucially, the observation of the impaired model elicited greater engagement of AOEN regions than the observation of the typically developing model, a finding consistent with the hypothesis that AOEN recruitment may be influenced by the similarity between observed kinematics and the child’s available motor repertoire. However, alternative factors such as attentional engagement, visuomotor mapping demands, or other task-related differences cannot be excluded, and the proposed role of kinematic similarity requires independent replication. The rehabilitation relevance of this principle was subsequently investigated in a randomized trial comparing conventional AOT based on observation of typically developing models with AOT based on motor-impaired models [18]. Children receiving the impaired model AOT showed greater short-term gains in upper-limb function, together with increased post-treatment recruitment of the ventral premotor cortex. These findings suggest that adapting the observed model to the child’s available movement repertoire may enhance both neural engagement and functional learning. One possible interpretation is that observed actions that are sufficiently compatible with the child’s available motor solutions may facilitate visuomotor mapping and AOEN recruitment more effectively than highly discrepant movement patterns. At the same time, the observed model may need to provide an achievable target for improvement rather than simply reproduce the child’s current impairment. This interpretation remains preliminary and requires independent replication.

Beyond the frontoparietal AOEN, action observation and imitation also engage cerebellar and subcortical circuits that may contribute to prediction, sequencing, and the updating of motor representations [2,75,76]. In healthy individuals, cerebellar activity and cortico-cerebellar connectivity have been linked to the accuracy of subsequent imitation [77]. In particular, activation of lateral cerebellar regions, together with premotor, parietal, and prefrontal areas, has been shown to predict imitation performance, while stronger functional coupling between cerebellum, anterior intraparietal cortex, and dorsolateral prefrontal cortex is associated with more accurate reproduction of observed actions. These findings support the view that the cerebellum contributes not only to movement execution but also to the generation and updating of predictive representations during action observation.

Basal ganglia may provide a complementary contribution through action selection, sequencing, reinforcement-based learning, and the consolidation of motor routines [78,79,80,81]. Together with cerebellar circuits, these structures extend the action observation–execution system beyond a purely cortical frontoparietal architecture. This broader network perspective may be particularly relevant in CP, where damage to cortical nodes, deep-grey structures, or their connecting pathways could alter the balance between cortical and subcortical contributions to action representation and learning.

Within a predictive-processing framework, action observation can be conceived as a continuous comparison between expected and observed movement trajectories and outcomes [76,82,83]. Sensorimotor priors, shaped by previous motor experience, may constrain the interpretation of the observed action, while prediction errors generated by mismatches between expected and incoming sensory information may contribute to updating internal action representations. In children with CP, atypical motor experience, altered structural connectivity, and reduced access to specific movement patterns could modify both the priors available for action prediction and the way prediction errors are integrated. This may be particularly relevant when the observed action differs substantially from the child’s own motor repertoire, potentially increasing the computational demands required to transform observation into an executable motor solution. These mechanisms remain largely hypothetical in CP and should be tested directly in future experimental studies.

From a rehabilitation perspective, cerebellar and subcortical circuits may also represent potential routes for compensatory reorganization when canonical cortical AOEN nodes or their connections are compromised [84]. This possibility is consistent with evidence from stroke showing that recovery following AOT may involve distributed cortical and subcortical circuits rather than a single frontoparietal system [84,85,86]. In children with CP, however, direct evidence for such compensatory mechanisms remains limited. It is therefore more appropriate to consider cerebellar and basal-ganglia recruitment as a plausible mechanism of developmental compensation that should be tested prospectively, rather than as an established feature of AOEN reorganization.

Taken together, current evidence indicates that action observation–execution mechanisms remain functionally recruitable in CP, although their organization may be altered according to early brain injury, corticospinal and network reorganization, and the child’s acquired motor experience. This reconfiguration may involve altered hemispheric balance, reduced differentiation of action parameters, and differences in responsiveness to movement patterns that vary in their similarity to the child’s own motor repertoire. Importantly, the system remains capable of experience-dependent plasticity and can support observational motor learning. These findings provide a rationale for investigating whether AOT outcomes may be improved by considering the child’s motor phenotype, lesion-related network organization, and ability to perceive, predict, and reproduce the observed action.

Key empirical studies directly informing current understanding of AOEN function, action observation, and AOT in children with CP are summarized in Table 1.

## 5. Neuropsychological Modulation of the AOEN

Although CP is primarily defined by disorders of movement and posture, the underlying early brain injury may also affect a broad range of neuropsychological functions, including attention, visuospatial processing, working memory, praxis, and executive control [22,88]. Neuropsychological functioning is highly heterogeneous across children with CP and may vary according to lesion timing, location and extent, motor phenotype, sensorimotor experience, and developmental reorganization. Therefore, children with comparable levels of motor impairment may differ substantially in their ability to perceive, retain, plan, and reproduce goal-directed actions. This variability is particularly relevant for rehabilitation approaches based on action observation, in which successful treatment requires more than the passive activation of sensorimotor cortical regions and may depend on the integrity of cognitive processes supporting action selection, prediction, sequencing, and observation–execution mapping.

On this ground, praxis represents a central interface between action observation and execution. It refers to the ability to organize and perform learned purposeful actions and depends on the integration of perceptual, conceptual, spatial, and motor processes [89,90]. Different forms of praxis impairment may affect distinct components of this transformation: ideomotor difficulties may primarily disrupt the translation of an action representation into the appropriate gesture or motor configuration, whereas ideational or conceptual difficulties may interfere with action goals, object-related knowledge, or the organization of multistep action sequences. To imitate an observed action, the child must identify its relevant features, understand its immediate goal, transform visual information into an appropriate motor programme, organize the movement in the correct spatial and temporal sequence, and monitor the ongoing response. Difficulties at any of these stages may result in inaccurate gesture reproduction, incorrect hand configurations, sequencing errors, or inefficient object use. Thus, a child may correctly recognize the general purpose of an action while remaining unable to translate that representation into an executable motor sequence.

This distinction is important for interpreting the relationship between praxis and AOEN functions. During action observation, frontoparietal regions contribute to the internal representation of the observed action. Successful imitation, however, additionally requires the selection, transformation, and adaptation of this representation to the child’s own body and motor capabilities [91,92,93]. Evidence from acquired apraxia indicates that action recognition, imitation, and object use depend on partially overlapping but dissociable neural networks [94]. Accordingly, poor imitation in CP should not automatically be interpreted as evidence of reduced AOEN recruitment or impaired action understanding. It may instead reflect a disruption in the higher-order processes required to transform an observed action into an achievable motor plan.

Executive and attentional functions represent a second major source of variability in AOEN recruitment and action-observation performance. This multidimensional construct includes sustained and selective attention, working memory, inhibitory control, cognitive flexibility, planning, and error monitoring [95,96,97]. These abilities are particularly important when the observed action is complex, unfamiliar, or composed of several sequential motor acts. Sustained attention is required to maintain engagement with the observed model, whereas selective attention helps prioritize task-relevant kinematic and goal-related cues. Working memory is required to maintain the relevant action components and their temporal order after the model is no longer visible. Planning supports their organization into a coherent goal-directed response, while inhibitory control suppresses competing, habitual, or compensatory movements that may be inappropriate for the task. Cognitive flexibility allows the child to modify an action strategy when the observed model differs from their usual movement pattern or when the first attempt is unsuccessful. Error monitoring is then required to compare the performed movement with the observed model and adjust subsequent attempts.

Additionally, attention is closely linked to these executive processes. During action observation, the child must remain engaged and selectively process the features that are most informative for successful reproduction. Depending on the task, these may include the effector involved, hand configuration, movement trajectory, object properties, temporal landmarks, or the final action goal. Attentional limitations may reduce the amount or relevance of information encoded from the observed action, particularly when several kinematic or goal-related features must be integrated. Impaired attentional shifting may also hinder the integration of successive movement components. Consequently, reduced imitation accuracy may arise even when the child retains a basic capacity to recognize the action and recruit corresponding action-related sensorimotor representations.

Moreover, visuospatial abilities are similarly relevant, particularly for actions requiring precise analysis of hand orientation, object position, trajectory, and spatial relationships between body parts. Mapping an observed action onto one’s own body requires transformations between allocentric and egocentric reference frames. These transformations may be especially demanding in children with CP because the observed model often has a motor repertoire and body configuration that differ from those of the observer. Visuospatial difficulties may therefore increase the distance between action perception and execution, particularly when the child is asked to reproduce complex object-oriented or bimanual actions.

Overall, the relative contribution of these neuropsychological factors may differ across CP phenotypes. In UCP, global cognitive functioning may be relatively preserved, although selective difficulties in executive control, visuospatial processing, attention, working memory, and praxis are frequently reported [22,27,98]. Emotional and behavioural difficulties, including problems with self-regulation, inattention, impulsivity, anxiety, or social participation, may also be present and can further influence engagement, adherence, and learning during rehabilitation [99]. The neuropsychological and behavioural profile cannot be predicted solely from lesion side, as developmental plasticity and network reorganization may partly compensate for focal damage. Nevertheless, asymmetric sensorimotor experience is likely to play a specific role. Reduced spontaneous use of the affected hand, altered somatosensory feedback, and compensatory reliance on the less affected limb may constrain the development of motor representations for unilateral and bimanual actions. At the same time, motor and cognitive development should not be viewed as independent or strictly unidirectional processes: the same early lesion may affect both domains, while reduced opportunities for motor exploration may secondarily limit perceptual learning, action representation, and the development of cognitive strategies relevant to observation-based rehabilitation.

In children with bilateral CP, including bilateral spastic phenotypes, neuropsychological difficulties may be broader because lesions and disconnections may involve bilateral periventricular white matter, thalamocortical pathways, and distributed frontoparietal networks. Difficulties in visuospatial processing, non-verbal reasoning, working memory, attentional control, and action sequencing may therefore coexist and jointly affect action observation and imitation [22,100]. Children with bilateral CP may recognize the overall goal of an action but could have difficulty in analysing its kinematics, maintaining its sequential structure, or transforming it into an organized motor sequence. However, these possibilities are supported mainly by the broader neuropsychological literature rather than by direct studies of AOEN function. Direct comparisons of AOEN functioning and action observation mechanisms between unilateral and bilateral CP remain scarce. These phenotype-specific distinctions should therefore be considered plausible working hypotheses rather than established neuropsychological models.

Thus, these considerations have direct implications for AOT. These neuropsychological differences suggest several candidate adaptations that could be tested in future AOT protocols. For example, reduced sustained attention might be addressed through shorter observation periods, fewer action components, or more frequent repetition; working-memory difficulties through shorter sequences, immediate execution, or repeated presentation of individual motor acts; and planning or flexibility difficulties through explicit action segmentation and progressive task variation. Praxis and visuospatial impairments might similarly motivate the use of simplified viewpoints, slower presentation, clearer object–hand relationships, or additional verbal and proprioceptive cues.

Thus, the same AOT protocol may impose substantially different cognitive demands across children, even when their motor impairment appears comparable.

On this ground, future studies should incorporate standardized neuropsychological measures and test whether specific cognitive profiles predict AOEN recruitment, imitation accuracy, and response to AOT. This approach would help determine whether individualized AOT protocols can be developed in which the model, stimulus complexity, sequence length, presentation rate, instructions, and level of repetition are calibrated to both the motor and neuropsychological profile of the child.

## 6. A Neuroconstructivist Framework for Observation-Based Motor Rehabilitation in Cerebral Palsy

The evidence reviewed above is broadly compatible with a neuroconstructivist interpretation of motor rehabilitation in CP (Figure 2). From this perspective, an early brain lesion should not be conceived as an event producing a fixed set of deficits. Rather, it can be understood as a developmental perturbation occurring while sensorimotor, cognitive, and social functions, including the AOEN, which progressively specializes across childhood, are still being developed. Its consequences are thought to emerge over time through reciprocal interactions among lesion timing and topography, structural disconnection, functional reorganization, bodily experience, cognition, behaviour, and environment. In neuroconstructivist terms, the child with CP can therefore be described not as a typically developing child with one selectively damaged system, but as an atypically developing system operating under different biological and experiential constraints [101].

The lesion introduces a discontinuity in the expected developmental trajectory, but it does not determine a single inevitable outcome. Children with apparently comparable lesions may develop different motor and cognitive phenotypes, as the effects of early damage are progressively shaped by the organization of preserved networks and by the experiences available to the child. This principle also applies to the AOEN, whose developmental configuration may diverge over time according to the interaction between lesion-related constraints, residual connectivity, and sensorimotor experience. Family interactions, opportunities for exploration and play, early education, rehabilitation intensity, social participation, and the demands placed on the affected limb can either constrain or support the emergence of new competencies. The phenotype observed at a particular age is therefore best understood as the temporary product of a continuing developmental process rather than the direct and immutable expression of the initial lesion.

Within this process, motor and cognitive development are unlikely to be considered independently. Attention, memory, visuospatial processing, praxis, executive functions, and behavioural regulation influence how the child explores the environment, selects relevant information, learns from others, and constructs increasingly complex action representations. At the same time, altered movement and reduced opportunities for autonomous interaction may limit the experiences through which these cognitive functions normally develop. From an embodied perspective, bodily action is not merely the final output of cognition but one of its developmental foundations [102]. Sensorimotor reorganization may therefore produce cascading effects on perception, language, executive control, and social learning. Conversely, interventions that increase the child’s capacity to act, explore, and interact also may potentially influence domains extending beyond motor performance [27].

Based on the available evidence, we propose three hypothetical functional profiles. These profiles are not intended as validated clinical subtypes, but as a heuristic framework for generating testable predictions about how structural organization, motor phenotype, and neuropsychological functioning may jointly influence action observation and treatment response.

(1) In a first profile, a child with relatively limited damage, preserved ipsilesional sensorimotor pathways, and no major involvement of frontostriatal or executive networks may retain a comparatively integrated action observation–execution system. AOEN recruitment may support the representation of both the overall goal of an action and its finer kinematic characteristics, potentially allowing the child to map actions performed by a typically developing model onto an accessible motor programme. Provided that attention, praxis, and executive functioning are sufficiently preserved, we predict that these children may show relatively efficient observation–execution mapping even when actions are presented by a typically developing model. This profile would therefore be expected to show a favourable response to conventional AOT, a prediction that requires prospective testing.

(2) A second profile may characterize children with greater motor impairment resulting from damage to critical sensorimotor or cortico-subcortical circuits, but with sufficient residual capacity for observational learning. Sanvido et al. [103] found that involvement of subcortical structures and lesion severity were associated with gross motor function in a cohort of children with dyskinetic CP, consistent with this pattern of damage providing indirect support for the relevance of subcortical damage to motor phenotype. Within our framework, we hypothesize that in children with substantial motor impairment but preserved observational-learning capacity, the discrepancy between actions performed by a highly proficient model and the child’s own motor repertoire may increase the demands of visuomotor mapping. Action perception may consequently place greater weight on the kinematic details required to construct an executable motor solution. A model displaying an improved but still familiar movement pattern may facilitate AOEN recruitment and observation–execution mapping by reducing the distance between observed and available actions. We therefore predict that, in this profile, AOT based on a model whose kinematics are closer to the child’s available motor solutions may produce greater learning than observation of a highly discrepant movement pattern [18]. Although this prediction is supported by preliminary neuroimaging and intervention findings, independent replication and testing across different CP phenotypes are required.

(3) A third, more severe profile may occur when extensive lesions and disconnections lead to marked contralesional or bilateral reorganization of the motor system. A recent fMRI study with children with unilateral CP reported that larger lesions were more frequently associated with contralesional motor reorganization and greater motor impairment [104]. Within our framework, such extensive reorganization may be associated with less differentiated observation–execution mapping. Shared control of the two upper limbs by overlapping neural populations may support broad representations of action outcomes while providing less differentiated coding of effector-specific, grip-related, or kinematic information. Such an organization could permit recognition of what an observed action is intended to achieve, while limiting precise mapping of how it should be reproduced with the affected limb. We therefore predict that these children may show a less efficient response to conventional AOT and may benefit from candidate adaptations aimed at reducing perceptual and cognitive demands. These could include simplified action sequences, multimodal cueing, or extended training, while virtual-reality environments may represent a promising platform for manipulating task difficulty and feedback. These adaptations remain hypotheses for future studies rather than established treatment recommendations.

Across all three profiles, neuropsychological functioning may alter the relationship between AOEN recruitment and rehabilitation outcome. Difficulties in sustained attention, working memory, praxis, visuospatial processing, or executive control may prevent the child from extracting, retaining, sequencing, and adapting the information conveyed by the observed action. These considerations suggest that AOT personalization should account not only for motor severity and lesion-related network organization, but also for the cognitive demands imposed by the protocol. Some of these parameters are already supported by evidence from the broader AOT literature in clinical populations, both children and adults. The complexity and number of motor acts appear to influence outcomes [15,105]. Regarding viewpoint, a first-person or egocentric perspective has been associated with more favourable observational-learning effects in some studies [106,107,108,109]. The use of virtual-reality-based stimuli has also yielded promising findings [84,110]. The comparison between live and video-based AOT remains less clear: some studies find no difference between the two formats [111], while others report better outcomes with live observation [112]. Evidence on presentation speed is limited to lower-limb AOT in stroke patients [113], with no data available for the upper limb. The duration of the observation phase has also been examined [114,115]. However, direct evidence for how these parameters should be adapted to individual neuropsychological profiles in children with CP remains limited. Other parameters, including treatment intensity, total session duration, and type of verbal instruction, remain largely unstandardized and underexplored, especially in CP, and should be regarded as proposed directions for personalization.

## 7. Future Research Priorities

The translational implication of this framework is a shift from diagnosis-based treatment selection toward developmental rehabilitation phenotyping. Accordingly, the central prediction of the proposed model is that rehabilitation outcome will be better explained by the interaction among structural and functional reorganization, motor phenotype, neuropsychological functioning, and stimulus characteristics than by any of these factors considered independently. If these profiles are supported by future empirical work, they could represent a useful framework not only for understanding individual variability in AOT response, but also for guiding the design of more targeted, individually tailored treatment protocols. Testing this hypothesis will require longitudinal multimodal studies combining structural and functional neuroimaging, quantitative motor assessment, neuropsychological testing, and systematic manipulation of the observed model. Larger and preferably multicentre studies should determine whether the proposed relationships generalize beyond the small and predominantly UCP samples that currently characterize much of the literature, including direct comparisons between unilateral and bilateral CP. Independent replication will be particularly important for hypotheses concerning model–observer kinematic similarity and the potential advantage of motor-impaired models. Future work should also directly compare the profiles proposed here against clinical and neuroimaging data to establish whether these profiles are empirically identifiable as distinct clinical and functional categories. Prospective studies should further manipulate candidate treatment parameters—including model type, task complexity, viewpoint, sequence length, and presentation characteristics—and examine whether cognitive and neurobiological measures act as moderators or clinically useful predictors of treatment response. Future studies should also examine whether fatigue and the energetic cost of action observation and subsequent execution influence learning efficiency and treatment tolerance, particularly in children with more severe motor impairment or reduced endurance. These factors should currently be considered potential modulators of AOT response rather than established determinants of outcome. Finally, studies should test whether matching AOT format and parameters to a child’s individual profile produces better outcomes than standardized protocols, which would provide stronger evidence for the clinical utility of this framework. If supported, this approach could move AOT from a largely standardized protocol toward a developmentally informed and genuinely individualized form of neurorehabilitation.

## Figures and Tables

**Figure 1:**
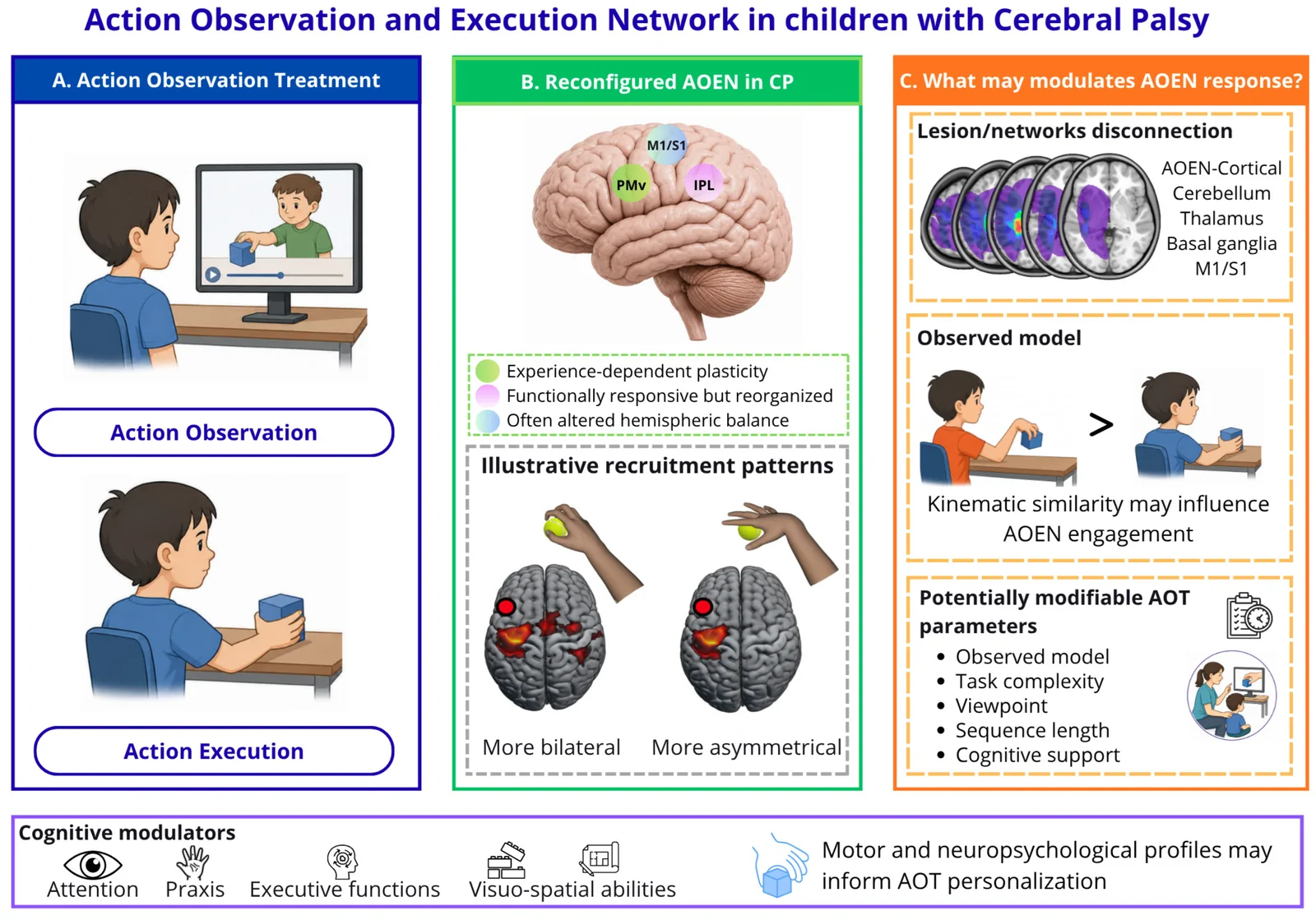
Action observation and execution mechanisms and their potential modulation in children with cerebral palsy. (**A**) Action Observation Treatment (AOT) combines the observation of goal-directed actions with their subsequent execution. (**B**) In cerebral palsy (CP), the action observation and execution network may remain functionally responsive but shows experience-dependent reorganization, including altered hemispheric balance and variable recruitment patterns. The examples shown are illustrative and do not represent fixed neurofunctional profiles. (**C**) AOEN recruitment and response to AOT may be influenced by lesion-related network disconnection, including cortical and subcortical structures, cerebellum, thalamus, basal ganglia, and sensorimotor regions; by characteristics of the observed model, including kinematic similarity; and by potentially modifiable treatment parameters such as model type, task complexity, viewpoint, sequence length, and cognitive support. Neuropsychological functions, including attention, praxis, executive functions, and visuospatial abilities, may further contribute to individual variability and inform future approaches to AOT personalization. Abbreviations: IPL = inferior parietal lobule; M1/S1 = primary sensory and motor cortex; PMv = ventral premotor cortex.

**Figure 2:**
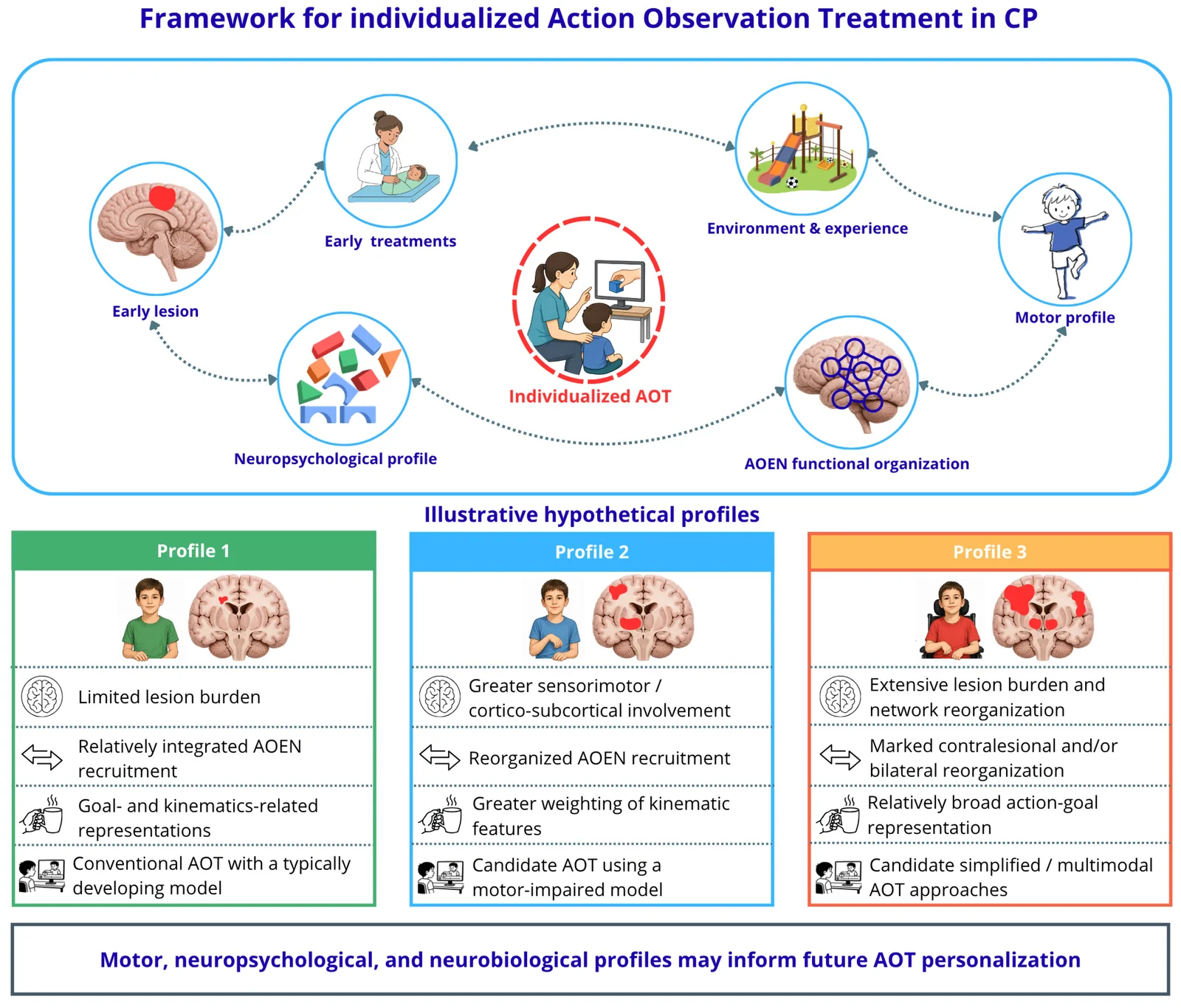
Framework for potentially individualized Action Observation Treatment in cerebral palsy. The upper panel illustrates the reciprocal interaction among early brain lesion, early treatments, environmental experience, motor and neuropsychological profiles, and AOEN functional organization in shaping individualized AOT. The lower panel presents three illustrative hypothetical profiles, ranging from limited lesion burden with relatively integrated AOEN recruitment and goal- and kinematics-related representations to greater sensorimotor and cortico-subcortical involvement with reorganized AOEN recruitment, and more extensive lesion burden associated with marked contralesional or bilateral reorganization and relatively broad action-goal representations. These profiles are intended as testable examples rather than validated clinical subtypes, and illustrate how motor, neuropsychological, and neurobiological characteristics may inform future AOT personalization, including the selection of the observed model and candidate treatment adaptations.

**Table 1:** Key empirical studies informing AOEN development, function, and Action Observation Treatment in cerebral palsy. Studies are presented chronologically. The table prioritizes empirical evidence obtained directly in children with cerebral palsy.

Study	Population/N	Method	Paradigm/ AOT Format	Main Finding	Key Limitation/ Interpretive Scope
Buccino et al., 2012 [87]	CP n = 15 (8 AOT, 7 control); 12 hemiplegia, 3 tetraplegia; age 6–11 y	Pilot randomized controlled trial	Observation of age-appropriate daily actions followed by imitation vs. non-motor video observation followed by execution of the same actions.	Children receiving AOT showed a significantly greater gain in upper-limb motor function than controls, providing early clinical evidence that observation–execution training may benefit children with CP.	Small pilot sample with mixed CP phenotypes; no neuroimaging or neurophysiological measure; clinical benefit does not identify the neural mechanism underlying treatment response.
Dinomais et al., 2013 [69]	UCP; n = 18 (age 7–21 y)	Task-fMRI	Observation of simple repetitive hand opening/closing performed by either hand.	Action observation recruited a broad bilateral occipito-temporo-parieto-frontal network, including premotor/parietal regions; M1 response varied with the observed hand.	Simple, non-object-directed action; heterogeneous age range and lesion characteristics; activation cannot be interpreted as direct evidence of AOEN activity.
Sgandurra et al., 2013 [16]	UCP n = 24	Evaluator-blinded randomized controlled trial	3-week AOT: observation of uni-/bimanual goal-directed actions followed by execution vs. motor practice after non-biological visual stimuli.	AOT produced greater improvements in upper-limb function than the control condition, supporting the clinical potential of observation–execution training.	Modest sample; clinical efficacy does not by itself identify the neural mechanism mediating improvement.
Buccino et al., 2018 [17]	CP n = 18 (AOT 11, control 7); fMRI subset n = 12	Randomized controlled trial + pre/post task-fMRI	3-week AOT using everyday arm/hand actions vs. non-motor visual control followed by matched execution.	AOT improved MUUL/AHA scores; treated children showed stronger post-treatment recruitment of a parieto-premotor circuit during object manipulation.	Small groups; fMRI available only in a subset; regional activation changes during execution cannot establish that increased AOEN recruitment causally mediated clinical gains.
Sgandurra et al., 2018 [9]	UCP n = 12; TD n = 12	Task-fMRI + TMS + clinical measures	Observation of simple vs. complex object-manipulation actions; active/passive sensorimotor tasks.	UCP showed greater AOEN lateralization toward the unaffected hemisphere and reduced modulation by action complexity; AOEN organization was related to motor-system reorganization.	Small sample; heterogeneous lesion timing/types; one participant excluded from one fMRI analysis for motion; representational interpretations remain indirect.
Errante et al., 2019 [12]	Right UCP n = 10; TD n = 10	Task-fMRI; subject-specific ROI/localizer approach	Observation of grasping performed by a typically developing model vs. a motor-impaired/pathological model.	Both models recruited AOEN-related regions; in UCP, the motor-impaired model elicited stronger parietal/premotor engagement, consistent with a role of model–observer kinematic similarity.	Small, highly selected homogeneous sample; only right UCP; no conventional whole-group GLM; kinematic-similarity interpretation requires independent replication.
Jongsma et al., 2020 [13]	UCP n = 15 analyzed; TD n = 45	EEG (sensorimotor mu rhythm)	Motor execution, action observation, and motor imagery for each hand.	Mu modulation in UCP was predominantly observed over the spared hemisphere, suggesting altered hemispheric balance of sensorimotor engagement.	Mu rhythm is an indirect/non-specific marker; small and clinically heterogeneous UCP sample; EEG topography may be influenced by lesion-related anatomy and source geometry.
Sgandurra et al., 2020 [71]	Congenital hemiplegia n = 8	Pilot randomized trial + longitudinal fMRI	3-week AOT vs. control visual material, with fMRI at baseline and follow-up.	AOT was associated with broader later fMRI recruitment and reduced lateralization of action-observation and sensorimotor networks, alongside functional improvement.	Small pilot sample; single-subject emphasis; findings are hypothesis-generating and require replication.
Beani et al., 2023 [73]	UCP n = 30	Randomized controlled trial; home-based tele-rehabilitation	Tele-UPCAT: intensive home-based AOT vs. standard care, followed longitudinally.	Home-based AOT improved bimanual function and dexterity, with effects persisting at follow-up, supporting feasibility and clinical effectiveness outside the clinic.	No direct AOEN neurophysiological measure; treatment intensity differed from standard care; does not identify which AOT component drove outcome.
Beani et al., 2024 [67]	UCP n = 35	Clinical structural MRI (MRICS + semi-quantitative MRI) + motor assessment	Association of lesion characteristics with MACS, BBT and mirror movements.	Greater lesion burden, particularly in the hemisphere contralateral to the more affected hand, was associated with poorer manual ability/dexterity; supports neuroanatomical phenotyping.	Cross-sectional association; no action-observation paradigm or AOT; structural measures are indirect with respect to AOEN organization.
Errante et al., 2024 [10]	UCP n = 16; TD comparison sample for structural analyses	Structural MRI, lesion mapping, VBM/q-BTS + task-fMRI	Characterization of synergic vs. semi-functional hand-manipulation phenotypes.	Manipulation profiles were associated with distinct cortical/subcortical and white-matter patterns; lesion localization was more informative than broad lesion timing, and functional lateralization differed across phenotypes.	Cross-sectional study; mild-to-moderate UCP impairment only; action observation was not directly tested.
Errante et al., 2024 [18]	UCP n = 26 (13 PAM-AOT, 13 TDM-AOT); fMRI subset n = 16	Evaluator-blinded two-arm RCT + fMRI subset	3-week AOT using a pathological ameliorative model vs. a typically developing model.	Both groups improved; PAM-AOT yielded greater short-term bimanual gains and greater post-treatment ventral premotor recruitment, supporting model characteristics as a candidate treatment moderator.	Single study for this research line; short-term between-group advantage; fMRI available in a subset; independent replication and testing in other CP phenotypes are needed.
Cruz-Sanabria et al., 2026 [68]	UCP n = 16	Longitudinal AOT cohort + semi-quantitative structural MRI	Home-based structured AOT with repeated AHA/BBT assessments; lesion–response associations tested longitudinally.	Temporal, thalamic, brainstem and callosal lesion involvement was associated with reduced improvement in selected motor outcomes, suggesting lesion architecture may contribute to AOT-response variability.	Exploratory observational analysis within an intervention cohort; small sample and multiple regional tests; candidate structural moderators are not yet clinically validated predictors.

Abbreviations: AOEN, Action Observation and Execution Network; AOT, Action Observation Treatment; BBT, Box and Block Test; CP, cerebral palsy; fMRI, functional magnetic resonance imaging; PAM-AOT, pathological ameliorative model AOT; TD, typically developing; TDM-AOT, typically developing model AOT; UCP, unilateral cerebral palsy.

## Data Availability

No new data were created or analyzed in this study. Data sharing is not applicable to this article.

## Abbreviations

The following abbreviations are used in this manuscript:

AOT	Action Observation Treatment
CP	Cerebral palsy
AOEN	Action Observation and Execution Network
UCP	Unilateral cerebral palsy
